# Perspectives of a mobile intervention for kidney transplant seekers: Post-intervention qualitative results from the *KidneyTIME* study

**DOI:** 10.1371/journal.pone.0325313

**Published:** 2025-05-30

**Authors:** Liise K. Kayler, Anne Solbu, Maria Keller, Matthew Handmacher, Ekaterina Noyes, John Von Visger, Thomas H. Feeley, Laurene Tumiel-Berhalter, Renee B. Cadzow

**Affiliations:** 1 Department of Surgery, Jacobs School of Medicine and Biomedical Sciences at the University at Buffalo, Buffalo, New York, United States of America; 2 Transplant and Kidney Care Regional Center of Excellence, Erie County Medical Center, Buffalo, New York, United States of America; 3 School of Public Health & Health Professions, University at Buffalo, State University of New York, Buffalo, New York, United States of America; 4 Division of Nephrology, Jacobs School of Medicine and Biomedical Sciences at the University at Buffalo, Buffalo, New York, United States of America; 5 Department of Communication, University at Buffalo, State University of New York, Buffalo, New York, United States of America; 6 Department of Family Medicine, University at Buffalo, State University of New York, Buffalo, New York, United States of America; De Montfort University, UNITED KINGDOM OF GREAT BRITAIN AND NORTHERN IRELAND

## Abstract

**Objective:**

Mobile health education could expand accessibility, but limited research has explored attitudes about such products among kidney transplant (KT) seekers. This study aimed to assess KT-seeker’s attitudes about the learning and donor search intervention in the *KidneyTIME* study, which examined the effectiveness of a web-based intervention leveraging animated video education, video viewing and sharing, and use-reminders compared to usual care video alone.

**Methods:**

Among 56 unique intervention-arm patients (54% non-Hispanic White, 58% no college degree, 46% total annual household income ≤ $30,000), we conducted semi-structured individual interviews with 31 patients and collected open-box survey comments from 36 patients. Using content analysis, we analyzed transcripts of the interviews to identify key concepts related to patient experience and usage of the mobile program as well as suggested improvements.

**Results:**

Most patients found the online program to be convenient and easy to access on any device, although a few recommended technological enhancements or availability earlier in the transplant process. The video content was helpful to learn and reduce anxiety about kidney transplantation and donation; additional topics were suggested and that it could be more personalized. Videos were shared to put others at ease, prepare them, and elicit possible donors. Some could have benefitted from sharing instructions or assistance or wanted other outreach modalities. The digital reminders to use the resource prevented forgetting and prompted watching.

**Conclusion:**

Patients had positive feelings about *KidneyTIME*, including receiving information from animated videos, sharing videos with their social network, and receiving email or text reminders. Findings provide insights about patients’ experience with this innovative approach for providing self-education and empowering patients’ social network outreach about kidney transplantation and donation, including future enhancements to consider.

## Introduction

Accessing kidney transplantation requires a kidney transplant (KT) seeker to know how to navigate the transplant process, find a living donor, and garner social support for transplantation [1]. Social network members often want to help KT-seekers, but don’t know how [2]. Transplant programs provide education, but it is usually offered infrequently and in person, limiting accessibility for many patients and their network. Mobile programs (websites, apps) offer the possibility of reaching patients and network members; however, it is unclear whether patients would independently use and share such programs [3,4].

We previously developed a mobile learning and donor search intervention, named ‘Kidney donation and Transplant Information Made Easy’ (*KidneyTIME*). The intervention is smartphone-optimized and leverages web-based animated educational videos and email or text messages to view and share them. *KidneyTIME* is designed to optimize living donor kidney transplantation (LDKT) based on our conceptualization of the information-motivation-behavioral skills (IMB) theory [1], which holds that helping patients and social network members meet three basic needs—feeling knowledgeable about the transplantation and donation process, feeling internally motivated towards receiving LDKT or donating a kidney, and having skills and confidence to conduct social network outreach-improves living donor volunteerism, social support for transplantation and donation, and transplant navigation. We also emphasized “ability” to conduct network outreach through simple support tools (sharing videos) in line with Bandura’s Social Cognitive Theory [5].

*KidneyTIME* showed feasibility to increase pre-post knowledge about LDKT by 71% [6] and is currently being trialed to compare the effectiveness of the intervention versus solely usual care video. The methods and main results from the trial are pending publication and will report the effects of the intervention on patient cognitions related to living kidney donation, patient transplant access behaviors, donor search, and living kidney donor inquiry. A mid-trial evaluation of intervention usage found that 74% viewed at least one optional video and 52% shared at least one study video over 6 months follow-up with no large differences across age, race, gender, and socioeconomic status [7]. At the end of the trial, we collected qualitative data from patients who were involved in the study to better understand and evaluate the intervention. Results will inform future refinement after multiple stages of user feedback. The purpose of this qualitative sub-study was to assess patients’ attitudes and experience with the mobile program and solicit suggested improvements.

## Methods

### Approach

To obtain more detailed feedback about the intervention in the *KidneyTIME* study, we conducted semi-structured phone interviews with KT-seekers randomized to the intervention arm between April 19, 2022 and December 8, 2023, and we collected participant comments to an open-ended survey question ‘Please provide us with any comments about the program that you would like to share.’ This study was approved by the University at Buffalo Human Research Institutional Review Board. The trial was also registered on ClinicalTrials.gov (#NCT05154773).

### Setting

This qualitative descriptive study took place at Erie County Medical Center (ECMC) in Buffalo, New York, a mixed urban-suburban city with a median household income of $48,904 and racial composition consisting of 44.3% non-Hispanic White, 34% Black, 12.1% Hispanic, and 9% Asian.

### Sampling

To be eligible for the original trial, patients must be referred to ECMC for a kidney transplant, English-speaking, age ≥ 18 years, have email or text access, and provide electronic consent. A total of 422 patients enrolled and 212 entered the intervention arm to receive the intervention and time-based surveys if they remained active in the study (i.e., eligible to receive a kidney transplant according to the transplant center selection committee). For the current study, we sought to obtain feedback from participants about their experience with the mobile program. We purposively recruited participants for interviews who were assigned to the intervention group and had completed the 12-month follow-up. We collected open-ended comments from intervention participants who completed a 1-, 6-, or 12-month survey and whose electronic data indicated they viewed at least one video on the interactive site.

### Intervention

**Fig 1:**
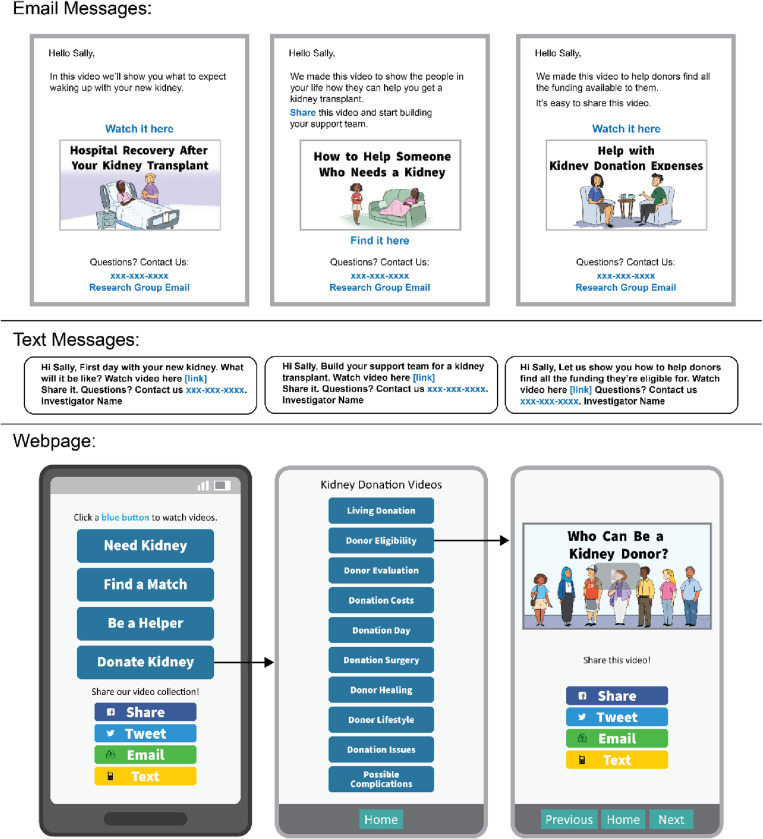
Intervention demonstration pages.

The intervention components (educational videos, web-interface, messages) were built to maximize accessibility for people with low literacy, low vision, and small devices [4,6,8,9] and are summarized in Table 1 with examples provided in Fig 1. The program content included kidney transplant and donation education targeted to potential recipients and donors within 25 animated educational videos, each 2–3 minutes long, specifically: 12 videos that describe the live kidney donation process, benefits, and risks, 1 video about transplant-process caregiving and donor search advocacy, 11 videos that describe kidney transplant process, benefits and risks, and 2 videos covering deceased-donor kidney allocation and the Kidney Donor Profile Index as alternatives to LDKT. The educational content implicitly includes caregivers and supportive others by showing their roles in all aspects of the transplant and donation process. All content and messaging within the study were written using clear communication strategies at or below an 8^th^ grade Flesh-Kincaid reading level by the research team, including experts in health communication, medical anthropology, clinical transplantation, and lived experience with kidney disease, donation, and caregiving.

**Table 1:** Components of the *KidneyTIME* intervention.

	*KidneyTIME*
**Core Animated Video Education about LDKT** (13 minutes)	Learn advantages of LDKT Learn opportunities of kidney exchange and donor financial support Learn general living kidney donor eligibility, process, surgery, outcomes.
**Optional Comprehensive** **Animated Video Education** (25 videos; total 55 minutes)	Learn more about kidney donation eligibility, process, surgery, and outcomes. Learn a wide variety of donor search tactics. Learn about kidney transplant eligibility, process, alternatives to LDKT, surgery, and outcomes. Learn about social network support opportunities from friends/family (donor search advocacy, caregiving) and professionals (finance, dietary).
**Animated Video Sharing** (25 videos)	Share educational videos about kidney transplantation and living donation, including using ubiquitous technologies (email, text, social media).
**Resource Access Messages** use-reminders (every 3 weeks for 1 year)	Receive links to website and messages about the opportunity to use the program and what a visitor may gain. Messages evenly cover transplant access and donation topics to address varying contexts, including 1 general and 12 unique messages.

LDKT, living-donor kidney transplantation.

**Fig 2:**
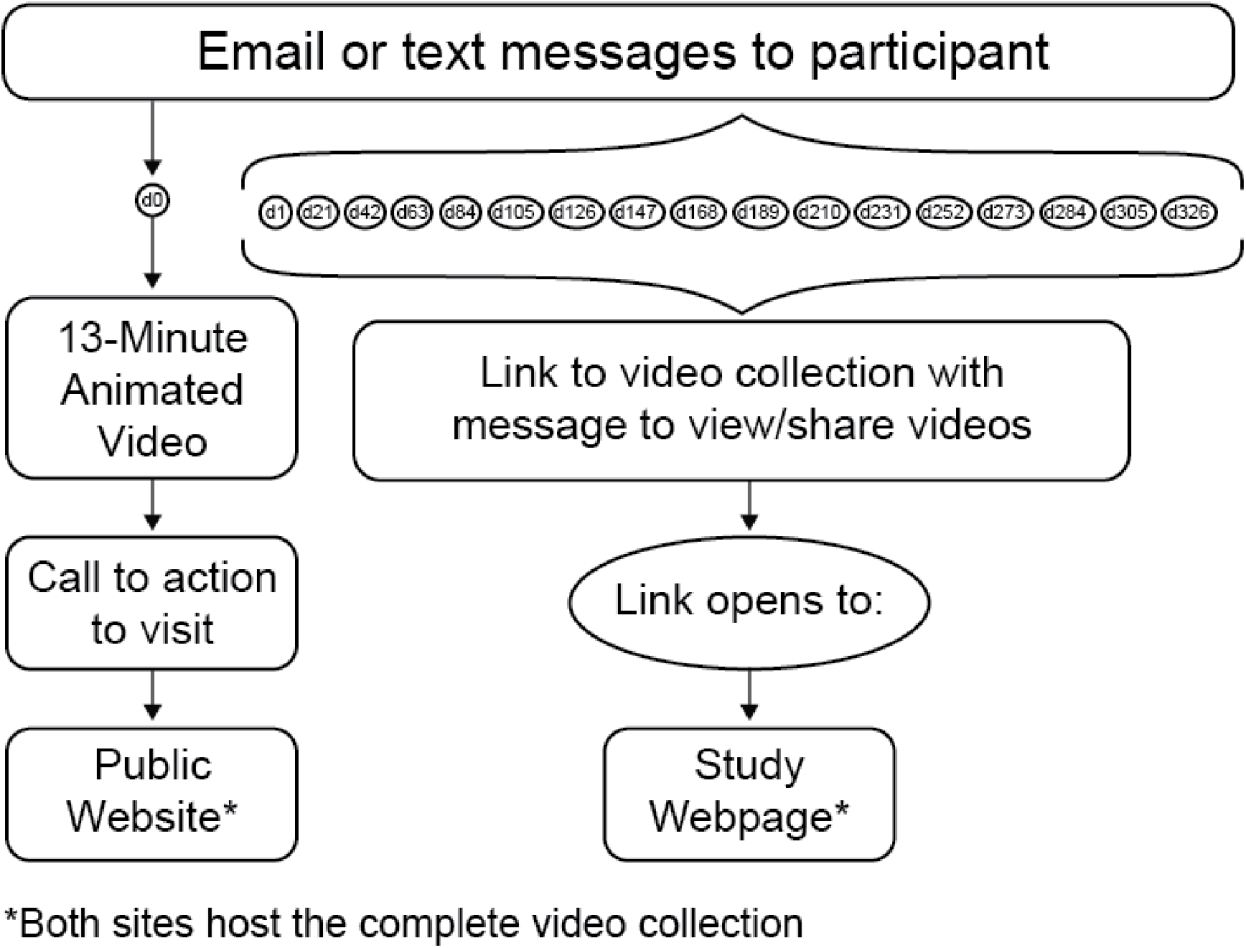
Study design.

As shown in Fig 2, patients in the intervention arm were sent a personalized link to open the web-based intervention via email or text without requiring log in. Intervention delivery included core education as a single 13-minute video emphasizing live donation (a collage of 6 videos), a study webpage and public website hosting the full video curriculum with more kidney transplant/donation information, and resource awareness messages with links to access the intervention videos (use-reminders). The videos were vertically listed behind 4 concept buttons requiring 3 clicks to access. Each video was activated for sharing using ubiquitous technologies (email, text, social media). The use-reminders, sent via email or text every 3 weeks for a year, evenly covered transplant and donation topics.

### Information collection

**Fig 3:**
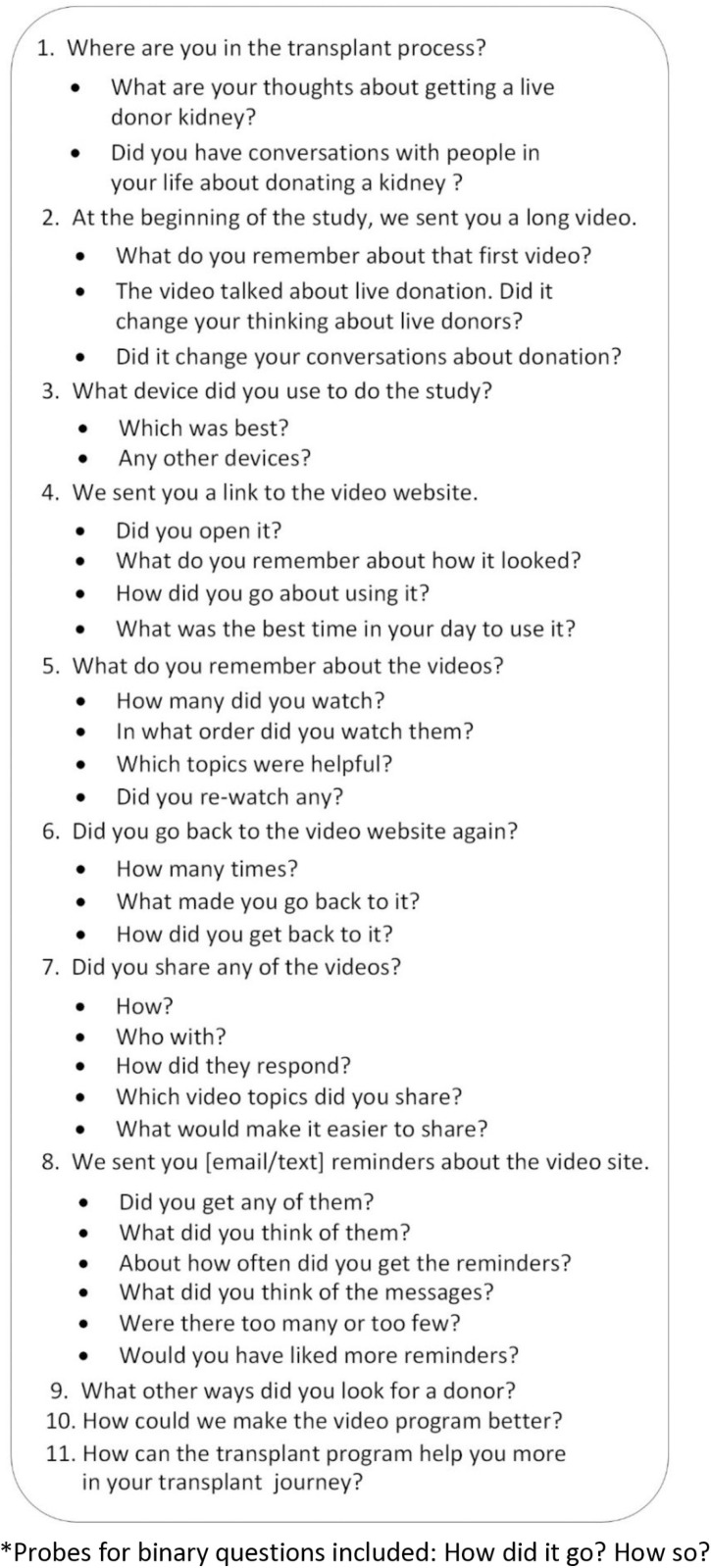
Interview guide*.

Patients who completed a final survey at 12 months (April 2023 and August 2024) received a question to assess interest in participating in a discussion about their experience in the study (n = 56). Those who responded affirmatively (n = 42) were provided an electronic consent form and after electronic signature followed up with a phone call a day later to describe the interview and confirm participation. Trained members of the research team (SP, CH) interviewed consented participants (n = 31) by telephone. Verbal consent to audio-record was confirmed. They used a semi-structured interview guide (Fig 3) to facilitate discussion and standardize data collection. The interview guide was developed based on the intervention components and expert opinion and piloted with several content experts, including our patient and stakeholder advisory committee members, to ensure clarity and appropriate length. The interview guide asked questions about the patient’s activities and experience with the online program, video shareability as the social network outreach enablement, program use-reminders, and related challenges and opportunities for improvement. Patients received a debit card for $25 after each completed survey and the interview to compensate for their time.

### Data collection and analysis

Of 122 participants who used the interactive site, 36 provided comments about the program at the end of a survey. Of 43 participants who completed the 12-month survey and expressed interest in an interview, 31 participants were interviewed. There were 56 unique participants since 11 patients contributed to both interviews and comments. The interviews lasted between 9 and 38 minutes and were recorded and transcribed. Transcripts and comments were independently read by the research team members (all white females), an anthropologist, two social scientists, and a transplant surgeon who developed an initial coding scheme. Analysis was both deductive, informed by the research objectives and interview and survey questions, and inductive, arising from multiple readings and interpretations of the responses [10]. The transcripts were imported into Dedoose (9.2.12) where one author, a doctoral-level social scientist, systematically coded each participants’ statements first deductively, by question topic, and then inductively into sub-concepts within those organizational categories. A second author, an anthropologist, reviewed the codes, reached consensus on any differences, and confirmed a final set of codes and subcodes. Reports were generated on categories (codes and sub-codes) within broad concepts of interest using general inductive approach [10,11]. While we did count code occurrence, emphasis was placed on range of responses and experiences, rather than occurrence frequency. The study team discussed the code reports, identified connections between them and derived cross-cutting concepts [10]. Participants were not involved in the analysis process or in confirming the accuracy of the transcripts and findings to avoid further burden of research. We also used descriptive statistics to report socio-demographics of participants from the intervention arm and the sub-study, showing clear representation of the wider population (Table 2).

**Table 2:** Baseline characteristics*.

Characteristics % (n)	Completed Interview (n = 31)	Provided Open Box Comment (n = 36)	Total Unique Participants (n = 56)	Intervention-arm sample in the RCT (n = 212)
Pre-evaluation phase at study entry	42%	61% (22)	54% (30)	51% (108)
Post-evaluation phase at study entry	58% (18)	39% (14)	46% (26)	49% (104)
Enrolled using email	61% (19)	53% (19)	55% (31)	64% (136)
Enrolled using text	39% (12)	47% (17)	45% (25)	36% (76)
Age < 50 years	32% (10)	25% (9)	27% (15)	31% (65)
Age 50–60 years	48% (15)	39% (14)	43% (24)	35% (75)
Age > 60 years	19% (6)	36% (13)	30% (17)	34% (72)
Sex, Male	39% (12)	53% (17)	45% (25)	56% (118)
Race, Black or African American	32% (10)	36% (13)	34% (19)	35% (74)
Race, non-Hispanic White	58% (18)	42% (15)	48% (27)	50% (107)
Race, Other	10% (3)	22% (8)	18% (10)	15% (31)
Education, less than college degree	52% (16)	67% (24)	59% (33)	60% (128)
Single adult household	32% (10)	33% (12)	30% (17)	28% (60)
Requires chronic dialysis treatment	71% (22)	67% (24)	71% (40)	77% (163)
Medicaid, state, or VA insurance	45% (14)	53% (19)	50% (28)	51% (109)
Employed	26% (8)	14% (5)	20% (11)	20% (42)
Total annual household income ≤ $30,000	48% (15)	47% (17)	48% (27)	48% (102)
Number of close friends or relatives 4+	55% (17)	50% (18)	52% (29)	47% (99)
Has working computer.	58% (18)	67% (24)	63% (35)	63% (132)

## Results

### Patient characteristics

**Fig 4:**
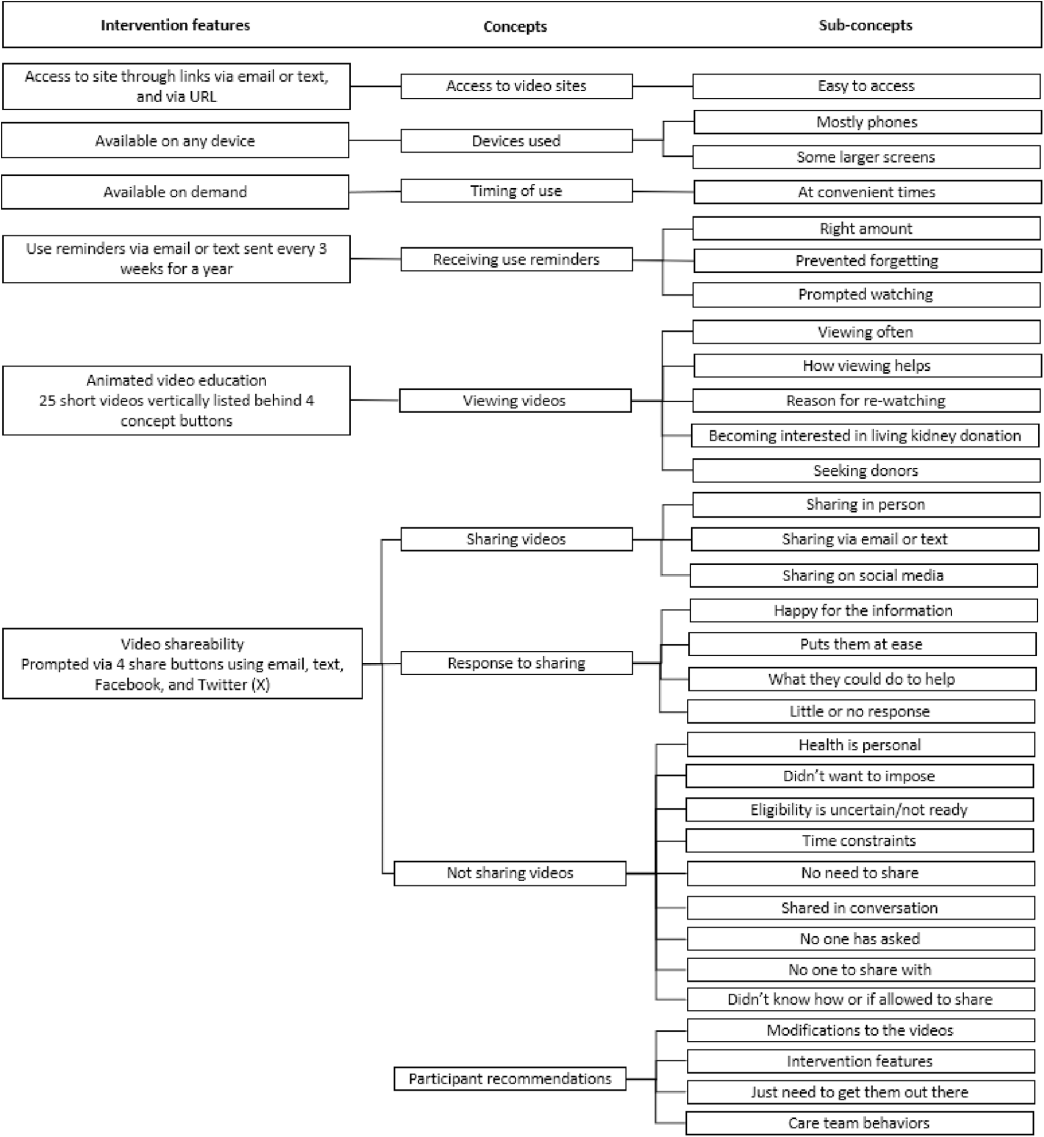
Summary of intervention features, concepts and sub-concepts of patient experience and engagement with the program.

Of the 56 unique patients who completed interviews or provided a survey comment, 54% were female, 54% were non-Hispanic white, 58% did not have a college degree, and 46% reported a total annual household income ≤ $30,000 at the time of the interview (Table 2). At enrollment, one-half of them were in the referral stage of evaluation. Study delivery was via email in 54% and text in 46%. As summarized in Fig 4, the codes aggregated into 9 concepts and 32 sub-concepts related to patient engagement and experience with the online program. Exemplar comments for each concept and sub-concept are provided in Table 3 and are summarized below.

**Table 3:** Representative quotes of concepts and sub-concepts of patients’ attitudes about the mobile program as well as suggestions for improvement.

Concepts *sub-concepts*	Representative quotes
1. Accessing the video interface	
*Easy to access*	*It was a text message. Every time I got a text message from kidney transplant, I would click on the link and it would take me right to the videos.* *I just kept the old link and just piggybacked off that one.* *One is the old email because that was handy. And another thing is once we open that video, it remains in the browser cache. So, if you type that, then it appears.*
2. Devices used	
*Mostly phones*	*It was really clear. I do have a computer but it’s very, very slow so I just decided to just use my phone.* *My iPad or my phone, whatever I was on.* *It was very convenient for watching on the phone because most of the time I’m active on phone rather than laptop or tablet.* *On my phone. That’s all I have.*
*Larger screens*	*The tablet because it’s much larger and I can see it more. It’s easier to look at it.* *I tried to open them on my phone and it does it, but just, you know, you got the nice big computer screen*
3. Timing of use	
*At convenient* *Times*	*When I got the text message saying the next link is up for the video, I watched them within a week*. *I didn’t open it up immediately, but I did open it up at some point. Me, even though I’m not working, I’m very busy.* *I like the fact that it was like on my own time and anytime I needed it I could go back to it.* *Phone’s always with me. I could watch them wherever, whatever I was doing.* *I would do it in the evening when I had time to sit down and concentrate and really listen without being disturbed.*
4. Receiving reminders	
*Right amount*	*It was the right amount of reminders* *I think, I mean, to me, a once a week reminder is enough, you know, and not too many cuz no one, you know, we get so much junk mail, it’s hard to, you know, go through.* *It tells me that I’m cared about that you wanna keep sending me this stuff.*
*Prevented forgetting*	*I would be so busy. I would forget about it and then I’d get a reminder and be like, oh yeah, lemme go and watch that one.*
*Prompted watching*	*I like when I get those, you know, because I took, I like to click in to see if it’s something new and, or it’s something that, even if I seen it before.* *I loved having the reminder. I loved watching the next thing.*
5. Viewing videos	
*Viewing often*	*It’s easy to watch. It’s not droll. You could pick the parts that you wanna see over and you don’t have to go through the whole thing to find it.* *It was a very easy study for me to just click on when I wanted to click on something.* *I watched them all in a row because they wasn’t that long.* *Once I finished with one link I just clicked on the next one and it just kept going.* *I could only break a part of what I was ready for at that time.*
*How viewing helps*	*I got most of all the information [from the transplant team] but it was like bits and pieces were coming in and bits and pieces weren’t. So, the videos helped fill in the spots.* *At times, it was really scary but having access to the videos and step by step, little by little about what was gonna be happening really put my mind at ease.* *Once I got that information to kind of rip the band aid off and when people ask question it was easy cause at first it sound stupid but I was like I felt ashamed about it. But then after getting the videos and realizing that a lot of people deal with this stuff it made it easier for me.* *When you think about the kidney process, it seems like such a mountain to climb, but these little chunks of information were like you could do this and then your donor does this you know? So, it just made it feel the process is not real cumbersome.* *After the video I could explain more to them about my kidneys.* *This is very helpful for both living kidney donor and kidney receiver. Please continue this program.* ^*^ *It allowed me to share accurate facts about the transplant process with my family. It provided a way to discuss this very tough subject and get answers together. After watching, we were able to have question answered during the evaluation.* ^*^
*Reasons for re-watching*	*For me it was being able to pause and go back if I had to and try to understand it. Make sure I totally grasped what I was seeing.* *I would be like, let me double check [after a question was asked], and then I’d go back.* *I’m broke. I need some money. So, if they’re going to pay me to watch these videos, then I’m gonna watch the videos.* *Just some things I would want kind of reiterated to me.*
*Becoming interested in living kidney donation*	*It actually changed my mind cause actually I wasn’t even thinking about living donation.* *I was completely against using a live donor due to the fear of having to worry about someone close to me. This program helped reconsider my choice.* ^*^ *It helped me take it a little bit more serious…now I really want to try to find a living donor.*
*Seeking donors*	*I liked knowing that I can answer people’s questions when they ask me.* *It did make me want to put myself out there.* *It opened my eyes to some other ways.* *The videos helped me understand the process a little bit better so that I could explain to people what the ask was and so that really helped.* *In the beginning [conversations] were harder because I didn’t have the videos yet.* *I never thought I would like reach out as much as I have until I watched that video.*
6. Sharing videos	
*S*haring in person	*At her dining room table, we were side-by-side where you can just sit down and listen to them.* *After my little brother decided that he was interested, then I went and watched the ones about the living donation with him so that we were both up to date on the stuff.*
*Sharing via email or text*	*I took the link from my email and copied it.* *I forwarded the email*
*Sharing on social media*	*When I put out the social media post, anybody that had questions, I would just copy the link and tell them to click that link and they could watch videos on the process.* *After one of the videos talked about using social media, I put out a post on social media and got 176 shares and probably about 50 people who were the same blood type as me willing to get tested*. *My kids helped me with the technology part. They taught me how to copy and paste and that’s how I sent them the videos.*
7. Response to sharing	
*Happy for the information*	*It went well. There were specific questions about what is the dialysis, I don’t understand the process, what is the process of needing a kidney, you know, things like that. So, it gave them the information they were asking for.* *My brother thought they were really good informative videos. Like I said he found out about the swap program through that.*
*Puts them at ease*	*I think it helped him be a little more at ease, too, because he was the same way he went on the internet and read all the things that aren’t necessarily true.*
*What they could do to help*	*I think that’s when we really started talking about it after the videos. When she really started thinking about it, when we saw the videos and it [donation] wasn’t gonna be such a hard.* *I shared with my sibling who stated the videos made him feel comfortable with being a living donor.* ^*^
*Little or no response*	*He didn’t really say too much. He just watched it and he said he didn’t really, he didn’t really gimme any feedback on it. We just, you know, watched it together. So, he didn’t really say too much.* *They would be right beside me. I’d say look at this. That would be it. They would not. No. No. Neither of them. No, they’re too busy. They’re not gonna look at the videos. They know everything.*
8. Not sharing the videos	
*Health is personal*	*I rarely let anyone know I am on dialysis and need a kidney. I feel not the need to put others in my medical business. I pray someday it will happen soon somehow.* ^*^
*Didn’t want to impose*	*I didn’t feel like watching a half an hour, 45 minutes to an hour worth of videos would be something that she would wanna do at that point after working all day.*
*Eligibility is uncertain/ not ready*	*I don’t wanna get somebody like all jazzed up to give me their kidney and then be like, oh no, I can’t. I’ll never get a kidney now.* *I will be sharing more and looking for a living donor in the next few weeks now that most of my testing is complete.* ^*^ *If he [the doctor] says something changed then I’ll be more putting it out there like if I have to put it on Facebook or something like that.*
*Time constraints*	*I think that would kind of be hard right now cause I’m on dialysis and I, it’s just a time restraint*
*No need to share*	*I have no problem sharing the videos except for I know everything there is. I know it all now.*
*Shared in conversation*	*I already shared the information in conversation.* *Just shared the knowledge. I didn’t personally give them the video to watch*
*No one has asked*	*I don’t know if I’d go outta my way to try and I don’t say force it, put the subject on them. But if they asked I would absolutely share it. I think it would be helpful for them.*
*No one to share with*	*Not a real reason. I guess I just didn’t have anybody to share ‘em with, really.* *Nobody really wants, you know*
*Didn’t know how or if allowed to share*	*I was thinking about sharing it with other people. Then I said, I don’t know how to get it off my email and show it to somebody else unless they’re here with me.* *I would love to [share on social media], but I don’t know if I was allowed to do it like that.*
9. Suggestions for improvement	
Modifications to the video	*They were done in a very positive note, and I’m not sure they revealed as much of the negatives that is really associated with transplant. Does that make sense?* *How do I ask family and friends to help me getting a kidney.* *I did wanna see more in depth of…the medicine that you take for it not to reject.* *Always have a second person. Even if it’s just a family member who decided to come with them that day. Someone else that can hear that information. ‘cause you will miss a lot of things if you, because you’re the person who’s going through it is going through so much.* *Highlight a success story, maybe give a little more positive light to it in a real person. There’s a difference between a cartoon character and a person and that connectivity.* *More stuff about a support system for folks in needs of transplant…have a short video about it and then at the end have links or resources.* *Single long video to reduce the need for clicking.* *Maybe split them* [videos] *up into smaller sections, some of them.*
*Intervention feature*s	*Put it in the subject line of the email: ‘This is highly recommended to be shareable.’* *Maybe more reminders to share it with people. Like for me, I’m not sharing it because I don’t know if I’m gonna ultimately be able to do this, but really I should be sharing it anyway because somebody else could benefit from that.* *Make it easier to share individual videos. We’re used to just a button that’ll say share.* *Like instruction how to share.* *An app so you don’t have to go looking for it every time.* *Have a search section so you can actually search what you were looking for*. *They really give you a green check mark to show what videos you already watched. The problem is that the green check mark that you get doesn’t stay on all of them.* *Just even a quick click of a button that says I wanna talk to somebody. I have a question, whatever it may be.* *Maybe questions and answers at the end or something.*
*Just* *need to* g*et them out there*	*The more you can get that, those videos out there, I, I think the better off it would be for anybody in this situation.* *Just keep on getting it out there. Maybe do a, uh, I know you guys do public service announcements and things like that, but maybe even do a show on it. A TV show.* *I would love to share it with people in the dialysis unit. I mean, they have signs up, up there and stuff like that, but I would try to make it easier for them. Uh, ‘cause everybody don’t know about it. Everybody don’t care, but a lot of people do.* *These videos, if they had been shown, like my doctor had access to them and could say, here I want you to watch these. I’m gonna hook you up with this site, it would have been so much better because it’s so much information at once that I can go back to it anytime I need to. And it would’ve been something that I could have kept referencing as my process changed.* *The best thing I could have done from the beginning of my journey if I could have those videos at that time.*
*Care team behaviors*	*And I just would encourage you guys to reach out a little more to patients.... instead of waiting for the end to do an exit interview, what about doing one halfway through?* *There should be some mandatory training for all this nice content because without mandatory, people will not do this.* *If there is any need for clarification, you* [transplant team] *can resend a video, you can tell them, oh, go look at this one again.* *I would like to see some kind of program where the person who doesn’t have a support system can still get a kidney.* *Better ways to reach people you don’t know. It’s hard to ask somebody to do something like that without knowing them. I think in your process something should be brought up as far as some type of advertising to help the people to get it or where to find out, whether it be a bumper sticker, a window cling, or a t-shirt or whatever, just to get the word out, you know, a sign for their front yard. There’s signs for everything.*

* Survey response.

(1) *Access to video sites.* Participants in general found it easy to access the webpage through links sent by email or text. Many respondents accessed the link through a saved email. Some mentioned that it was stored in their browser. None indicated that they typed in the URL for the website.
(2) *Devices used.* Nearly all interview respondents indicated they accessed the videos from their phone (n = 25). This was either due to convenience or because that was the only device they had. Seven also mentioned they used their computer, often for a larger picture. Phones were reported as being more “convenient,” “faster,” and “reliable.”
(3) *Timing of use*. Patients generally emphasized the convenience of on-demand access. Most used the online program early after receiving access to it. A few used it more later in the study when they had time to “take in the information.” Times that they viewed the program were “after the kids went to bed,” waiting at “doctor’s appointments,” “during dialysis,” and at the “bus stop.” Two patients would have liked availability of the program when they “first started” the transplant process.
(4) *Receiving use reminders*. Nearly all participants reported that the number of reminders was appropriate or sufficient and that they were very helpful. The reminders helped them remember the resource and prompted watching. Most recalled getting reminders between once a month and twice a month. Some saved the reminders for later viewing. One participant indicated that the reminders made them feel cared for by the transplant team. Another thought a few more reminders would be good. Others cautioned against doing too many reminders (it would inundate their email or make them feel pressured).
(5) *Viewing videos*. In terms of program functionality, patients found the site to be well structured and simple to use by clicking on videos of choice. Many patients reported viewing all the videos, usually in a row, whereas others viewed a few at a time. Some mentioned re-watching videos and visiting the site multiple times. Reasons reported for re-watching included “refresher/make sure didn’t miss anything,” “in case someone wants to talk about it,” because they weren’t sure “if they watched them yet” (confusion with the technology), and because they (erroneously) thought they would receive compensation for viewing. The information in the videos generally helped them understand the kidney transplant process; they were particularly focused on videos related to the living donation process and transplant recovery. Some reported that the video information made them able to talk more confidently with others about kidney transplantation. One reported that the information alleviated shame about their condition. The majority stated that the videos confirmed what they were already thinking regarding living donation, either already interested or not interested. Non-interest was generally due to concern for donor health or perceived unavailability of donors. Some stated that the videos changed their mindset and actions towards living donation. Patients mentioned starting to think about donors, feeling more comfortable asking and explaining, learning different strategies for finding donors, and doing more outreach behaviors. Information considered important to discuss with potential donors was “they can live a full life after,” “you don’t have to pay out of pocket,” and “my insurance would help cover.”
(6) *Sharing videos*. Nineteen interviewees said they shared the videos in some way, while 12 did not share the videos. Regarding how they shared them, participants responded that they shared them in-person, via email or text, and sometimes social media. Participants said they would often watch the videos together with family or others on their phone while sitting side by side at a table, on the couch, or the edge of a bed. When they shared via email or text, they copied and pasted the link into a text, forwarded the email, or sometimes used the share button feature on the site. They often shared the link to the whole library of videos. A few participants shared the videos on social media—mostly Facebook, but also Instagram and WhatsApp. Two respondents intend to post on social media but had not yet at the time of the interview. Some mentioned needing technology support from their children to share the videos. Respondents mostly shared the videos with family and people close to them and less often in their wider social network. Respondents often indicated why they shared them; the most common reason was to help their loved ones understand the process. A couple of respondents also indicated it was a way to elicit possible donors.
(7) *Response to sharing*. In line with the participants’ intentions for sharing the videos, participants said that their friends and/or family were “happy for the information,” found it “informative” and “good” and that it answered questions and “made it clear to them what exactly was going on with me.” A couple of participants described their family as more at ease after watching the videos. Four respondents talked about how their family or friends asked what they could do to help, offered to get tested, began researching the donor surgery process, and looked into the kidney exchange program. Several indicated that there had been little or no response to the shared videos. In one situation, the video likely reinforced information that the family member already had received.
(8) *Not sharing videos*. More than a third of the respondents reported not sharing the videos with others. Reasons included “already shared the information in conversation,” “no one to share them with,” “no one has asked,” “health is personal,” “didn’t want to impose,” “not ready yet,” and “didn’t know how.” The most common responses were that health is personal and they didn’t want to impose. These responses, along with not being ready yet, alluded to the uncertainty about eligibility for a transplant, which was explicitly stated by a couple of respondents who were waiting to hear if they would be put on the waiting list or felt they had time because they were not yet on dialysis. One participant expressed that people have their own issues going on and they don’t want to add to them. They may have been more likely to share their needs if they were younger, but given that their network is all older, they don’t want to add to their many problems. Some said they weren’t ready to share yet, implying that eventually they may be willing to share with others, perhaps when their eligibility is clearer or there is greater urgency.
(9) *Participant recommendations*. Participants shared recommendations related to the intervention, the care team, and the need to increase awareness and communication about kidney transplantation. Many respondents mentioned specific modifications to the video content or length. Specific content suggestions included: more information on the financial considerations and funding support (including health insurance), specific information about donation (finding a donor), caregiving, transplantation (more on medications), and dialysis, include negatives – like how much pain you will experience, and adjust video length (too short, too long). Participants also had suggestions related the features of the current intervention – make the videos easier to share and emphasize sharing more, provide sharing instructions, have a link to a live chat or a number to call with questions, create a search option, create links to other regional supports and resources, and ensure that the videos that have already been watched are clearly marked. Some respondents stated that the videos were great and that ‘they just needed to get them out there’ – through social media, billboards, in person sessions at dialysis centers, and in newspapers. Participants also suggested modifications to the care team behaviors, including checking in with the patient more frequently, providing more support in finding a donor, and making the videos mandatory for being added to the transplant list.

## Discussion

In this qualitative study, we conducted semi-structured individual interviews with patients pursuing kidney transplantation who were involved in the *KidneyTIME* trial to obtain more detailed feedback about the intervention and to help understand how to improve it from the patient perspective. To our knowledge, *KidneyTIME* is the first study for kidney transplant access evaluating a mobile intervention that is completely remote and self-directed by patients. Worldwide, there is a high need for interventions tackling kidney transplant access behaviors at the individual and social network level. An important medium for reaching a high proportion of the population with interventions is the internet, specifically websites and downloadable or social media apps. Few such evidence-based products exist with the goal of activating transplant access [12–16], and there is limited research that has explored issues around sustaining participation in these types of programs. To maintain interest over time, we provided barrier-free access, availability on any device anytime, kept the program simple and user friendly, and emailed/texted reminders that had positive and supportive messages and links to use the intervention. In this study, we learned from participants the utility of the program and unmet needs in terms of access, using the site, and program enhancement suggestions.

### Program utility and unmet needs

The *KidneyTIME* program could be opened through unique links sent via email or text and was reported to be easy to access. It did not require logging-in, entering time-consuming data fields, or downloading an app. This approach was based on our previous research that found that requiring log on was a barrier to use in 16% of the population referred to our transplant center [17]. To ensure access, a URL to a publicly available version of the site was promoted at the end of each video, but participants did not mention using it or knowing of its availability. Some participants recommended dissemination of the intervention from their provider. As such, the URL may become more useful later for post-trial clinical integration of the intervention into the real clinic; however, new strategies will be needed to enable staff to disseminate the URL and motivate uptake of the resource by individuals.

In terms of using the site, participants liked the convenience of on-demand availability, and most felt the site was well structured, simple to use, and helpful. Patients generally liked using their phone to access the online program; however, some preferred a larger screen. Accessing LDKT education through phones may be important to ensure broad reach since patients with less education, lower incomes, and racially or ethnically minoritized patients have lower access to computers [16,18] and the fastest increases in cell phone adoption [19]. Some participants requested more features, such as a search option, and the opportunity to stop at any moment and proceed at a later time without information about earlier viewing being lost, such as through a downloadable app. Findings reinforce that future modifications of the intervention should maintain the simple hierarchy of the original site structure. Future requirements to log in would allow past viewing activity to be saved, but may be a barrier to access.

The video content was considered helpful for learning and reducing anxiety about kidney transplantation and donation, both among patients and their sharees. The most popular topics were about living kidney donation and transplant recovery; these qualitative findings are corroborated by electronic video viewing data (reported elsewhere) which showed that the top three most viewed videos were on the basics of living kidney donation, complications of living kidney donation, and recovery after transplantation [7]. For some, the content increased interest in living donor kidney transplantation, comfort and motivation in asking for donation, as well as doing other donor search actions. Several wanted more topics or detail. Some would have liked information reinforcement, such as through post-video questions and answers; personalization, such as the ability to ask questions; or video recommendations tailored to their informational needs.

Although most accessed the program immediately, a few participants expressed use challenges, citing timing, forgetting, and lack of availability (poor health) as reasons why they were unable to use the system when it was first sent to them. The reminder messages helped to overcome forgetting and prompted usage, especially when the message highlighted previously unseen videos. These findings are consistent with digital usage data showing that 48% of users opened the webpage in the first month of intervention webpage access. At 6-months follow-up (whereby participants had received 8 reminders), intervention usage increased to 74% [7]. In line with engagement research [20], the electronic reminder messages were designed to stand out and to make them generally relevant (to KT-seekers) and persuasive by describing what a person might gain by viewing or sharing the content. We were mindful of not burdening respondents with too frequent reminders. As there are no data in the literature on digital use messages to guide the correct dose in the kidney population, we relied on our community partner’s advice prior to the intervention to set the dose of 17 total messages over 1 year. Patients reported that the number of messages was sufficient or could have been more frequent and made them feel we cared. Only 3% (6/195) of intervention participants opted to stop receiving electronic messages, suggesting that message fatigue was not an issue.

Patients reported that the option of video sharing was useful, as it enabled them to easily disseminate education to others, sometimes without having to explain it themselves. Most shared videos to close contacts and in person (side-by-side), rather than asynchronously. Trial data corroborates that 52% shared the intervention in the first 6 months [7]. The videos were shared to put others at ease, prepare them, and elicit possible donors. People’s responses to the shared videos generally included feeling happy for the information and interested in helping. Several, however, indicated that they had little to no response after sharing the videos. Limited sharing was sometimes reported due to not being willing or ready to find a donor, not wanting to impose on others, or not knowing how to share. Some patients requested instructions for sharing videos and greater emphasis on sharing in messaging. We attempted to make the site functionality intuitive; however, some participants could have benefitted from sharing instructions or assistance. Although video shareability enabled patients to disseminate LDKT information, the heterogeneity in sharing behaviors shows that there is room for improvement. To further promote intervention usage and donor search, additional components of the intervention may be needed to lower the barriers for patients to engage in the desired action (bumper sticker, t-shirt) or further enabled by providing human facilitation, such as mobilizing caregivers, involving navigators or leveraging provider encouragement to take the action.

### Comparison to prior studies

Other kidney transplant access interventions that provided digital tools for participant-directed use have provided websites [13,16], tablet computers [14], and mobile or social media apps [12,15]. Participant experience using the provided digital tools is sparsely reported. After viewing a multimedia website at the transplant center, 33% of participants viewed it after returning home with nearly half viewing it together with others [16]. Based on a 3-week follow-up telephone survey, participants expressed positive attitudes about the use of the website (in terms of understandability, ease of use, plan to revisit, and would recommend to other patients and friends/family) [16]. The most popular sections were about living donors and financial issues. A study that gave participants the option of downloading a mobile app containing animated videos and a risk calculator did not detail participants experience or extent of usage [12]. A study that mailed tablet computers to participants homes reported connection issues and usability barriers and 70% viewed the study videos on the tablet [14]. In a Facebook app study for posting personal stories and sharing links, only 17% of participants allowed the researchers to view their postings, so the extent of intervention uptake is unknown [15]. The *KidneyTIME* intervention made use of participants’ own devices and email/text messaging to provide web-based smartphone-optimized content. Based on a prior quantitative study [7] and the current qualitative study of intervention usage by participants several insights can be drawn. Interventions using email or text messages are low burden in that receiving intervention messages requires no user effort and allows patients flexibility to participate through their phones on their own time. Design techniques to encourage revisiting may include not requiring logging in or downloading an app. Use of automated messages to prompt intervention usage may have additional benefit to prevent ‘forgetting’. In terms of reach, intervention uptake data previously found similar rates of visiting the intervention by disadvantaged populations [7], suggesting that it is useful for dissemination to these populations.

### Limitations

This study has several limitations. The study was conducted at a single institution and only among English-speaking adult patients referred to a transplant center with email or text access who were predominantly Black or White race; therefore, the results may not be transferable to other settings, contexts, or populations. We restricted the interviews to patients in the intervention group, who had completed the 12-month follow-up survey. This sampling method excludes patients who had low engagement. We wanted to focus on those who had been most engaged in the study in order to get more detailed feedback about the intervention that would be helpful for future improvements.

## Conclusion

The results from this study highlight the potential value of mobile education-outreach interventions in the pre-transplant setting. Patients had positive feelings about the intervention, including information support in the form of animated videos, outreach enablement from video sharing, and use-reminders. Future work will use insights from this study to investigate how to improve the intervention for maximum patient engagement and outcomes.
